# Does carnitine supplementation truly increase whole‐body fat oxidation in older male adults during moderate‐intensity exercise?

**DOI:** 10.1111/acel.13449

**Published:** 2021-08-12

**Authors:** Dumitru Constantin‐Teodosiu

**Affiliations:** ^1^ School of Life Sciences University of Nottingham Medical School Nottingham UK

## CONFLICT OF INTEREST

The authors declare that they have no conflict of interest.

Chee et al. (2021) have recently claimed that supplementing older male adults (70 years; *n* = 7, 3 of whom were on statins medication) with a daily carnitine and protein formulation that also contained 44 g of sugars in conjunction with twice‐weekly exercise training sessions at 50% VO2max over 25 weeks would increase their muscle total carnitine stores. Subsequently, the higher muscle total carnitine levels were hypothesised to (1) improve whole‐body insulin sensitivity and (2) increase whole‐body fat oxidation during a moderate‐intensity exercise undertaken after training. The reference was made against a similar age and medicated small group of male adults who received a similar drink formulation (but without carnitine) and exercise training.

The authors acknowledged there was no basis for upholding the claim that carnitine supplementation would improve resting insulin‐stimulated whole‐body or skeletal muscle glucose disposal. Furthermore, the authors could not show any significant mean group differences in energy expenditure, nor in the rates of plasma appearance or disappearance during two supposedly identical exercise tests—1 h of exercise at 50% VO2max—undertaken before and after carnitine/placebo supplementation. Conversely, the authors upheld the claim that carnitine supplementation in older male adults would increase whole‐body fat oxidation, predominantly in the form of intermyofibrillar lipids (IMCL). Nevertheless, the evidence called in to support the latter claim does not stand up to detailed scrutiny, is circumstantial at best or is missing, and may convey an unsubstantiated message to the public. In fact, the personal interpretation of the present commentator, which will be detailed later, is that carnitine supplementation in conjunction with bi‐weekly training sessions for 25 weeks increased CHO oxidation rather than that of IMCL.

Let us assume for a moment that the authors’ claim that a 20% rise in fat/IMCL oxidation would occur with carnitine supplementation and training is correct. Then, an extra ~40 (220–180) J/kg lean body released from fat/IMCL would have contributed to the energy expenditure in the carnitine treated group during the 60 min exercise test at 50%VO2max. Also, assuming a total lean mass of 50 kg (table 1; Chee et al., 2021) and that 1 g of triacylglycerol generated 39.4 kJ through oxidation (authors’ conversion factor), then an additional (40 × 60 × 50)/39,400 or 3 g of fat/IMCL would have been burnt during each exercise session. However, an increase in fat oxidation in the treated group should have occurred earlier rather than exclusively during the exercise test undertaken at the end of the training. If we assume generously that the additional fat oxidation with carnitine loading started from the first week of training, then a total of 150 g of fat/IMCL (3 g fat × 2 sessions per week × 25 weeks) would have been oxidised over 25 weeks (or <1 g fat/daily on average). In line with these calculations, the data displayed in table 1 and figure 5d (Chee et al., 2021) show no change in any regional fat content across all subjects irrespective of group or time. Equally, this minute amount of fat, which could have certainly not been captured by a DEXA scan, would have also been easily masked by the effects on the whole‐body composition by the additional 44 g of sugars that all subjects had to ingest daily for 25 weeks. Overall, the claim that total carnitine would increase fat oxidation by 20%, predominantly in muscle IMCL, during the exercise test would have been insignificant when translated to an absolute value. It is also worth remembering that the reported increase in fat oxidation during the exercise test was derived from data recorded from a male cohort where six out of fourteen were on statins medication, a drug well‐known to interfere with whole‐body fat handling.

The males in the treated group appeared to store primarily 22% more muscle total carnitine than in the control group, even before supplementation (figure 1a; 3rd vs 1st column; Chee et al., 2021). However, a control male with the lowest muscle total carnitine content (10 mmol/kg dm) of all males enrolled in the study may have contributed to the sizable difference between the groups at the baseline. At the end of the training, it was equally unexpected to notice a marked decline in muscle total carnitine once again in another control male (figure 1a, left panel; Chee et al., 2021). Given the small number of males in each group, these two control males were, therefore, most likely to have acted as leverage points to biasedly increase the mean difference between the treated group and control at 25 weeks, thereby raising the chance of declaring a false‐positive finding (figure 1b; Chee et al., 2021).

The authors also state that the values of the carnitine forms reported in the study cover the main three forms of carnitine: free, short‐ and long‐chain acylcarnitine. However, as figure 1 (Chee et al., 2021) demonstrates, the reported values in the published paper represent free and acetylcarnitine values only. Therefore, the contribution of the long‐chain acylcarnitine form, which is not trivial, is missing.

From a biochemical perspective, the present assertion that carnitine supplementation could have contributed to an increase in fat/IMCL also seems to be in collusion with *in vitro* experiments, which have reported that the Michaelis‐Menten constant (*K*_m_) of CPT1—reputed to be the main limiting step in fat oxidation—for free carnitine is 0.4 mmol l^−1^ (McGarry et al., 1983). Since the lowest muscle‐free carnitine content reported in the present study (~10 mmol kg^−1^ dm or ~4 mmol l^−1^ intracellular water; values computed from figure 1b; Chee et al., 2021) was well above (~10‐fold) the reported *K*_m_ value for free carnitine, this would have made the muscle CPT1 enzyme kinetics independent of that of carnitine concentration. Therefore, any further increases in muscle‐free carnitine would have been irrelevant to achieving the maximum capacity of muscle to oxidise fat.

The authors seemed to consistently and unjustifiable assign changes in muscle total carnitine, rather than free carnitine, the leading role to support their upheld hypothesis. This consideration is unwarranted since it is the free carnitine form that facilitates the transport of long‐chain acylcarnitines across the mitochondrial membrane. Equally unjustifiable was conveying the belief that the calculated increase in whole‐body fat oxidation could have been accounted for by a rise in muscle IMCL oxidation. It was not surprising that the muscle‐free carnitine mean differences among groups or the main effect of free carnitine on muscle IMCL use were not statistically different (figure 1 and table 2, respectively; Chee et al., 2021), and the amount of additional IMCL burnt during the exercise test post‐carnitine supplementation was indeed so negligible.

It is interesting to note that obese people (Harper et al., 1995) and chronic high fat dietary intake (Constantin‐Teodosiu et al., 2019), conditions that are associated with an increase in circulating insulin levels to make up for an increase in insulin resistance, are inherently associated with higher total muscle and liver carnitine levels compared with subjects who are leaner. However, it remains to be established whether the natural rise in muscle‐free carnitine stores with increased fat availability is due to (i) a cellular response in the face of an increase in fat handling or (ii) due to the higher content in free carnitine by the dietary fat or in combination with the higher‐than‐normal insulin levels.

The rate of muscle acetylcarnitine accumulation is a well‐accepted marker of the flux through pyruvate dehydrogenase complex (PDC) reaction, the enzyme that limits the rate of carbohydrate oxidation (Constantin‐Teodosiu et al., 1991). The most remarkable change in the pre–post training muscle acetylcarnitine accumulation was recorded in the treated group (figure 1b, right panel; Chee et al., 2021). Additionally, the lowest difference in the pre–post training muscle lactate accumulation, a well‐accepted anaerobic metabolism marker, was also recorded in the treated group (figure 3c; Chee et al., 2021). In line with the latter observation, the lowest blood plasma levels, a marker of the amount of lactate produced and released by contracting muscle, were recorded in the treated group (figure 4b; Chee et al., 2021). Collectively, these data would suggest that the flux through PDC reaction in the treated group post‐training was the highest of all conditions.

All in all, the results of three straight biochemical measurements (increased rates of muscle acetylcarnitine accumulation matched by reduced rates of muscle lactate accumulation and circulating blood levels) along with a significant increase in the respiratory exchange ratio during the hyperinsulinaemic‐euglycemic clamp (RER; figure 5c; Chee et al., 2021) point to an increase in the oxidative CHO contribution, rather than that of fat, to the total energy expenditure in the carnitine treated group with training. Despite this firm evidence, along with the finding that energy expenditure during exercise was similar across treated/control groups (figure 2a; Chee et al., 2021), the authors claimed that more fat/IMCL was oxidised in the treated group than in the control during the exercise test. This assertion/calculation relied exclusively on the results of a multi‐step convoluted gas chromatography mass‐spectrometry (GA‐MS) method.

The post‐/pre‐training fold changes in gene expression of selected muscle transcripts involved in fatty oxidation (ACAT1) and IMCL turnover (DGKD and PLIN2) were significantly greater in the carnitine group than in the control group (table 3; Chee et al., 2021). However, the reported differences, albeit significantly different, were vastly low to assign them a physiological significance. Interestingly, the muscle‐specific CPT1B isoform gene expression in the control group was higher than in the carnitine group (table 3; Chee et al., 2021). This finding would advocate for better FFAs handling in control males than in the carnitine treated males. In addition, this observation would be another critical point at undoing the authors’ argument that only carnitine supplementation, rather than training per se, could increase the mitochondrial fatty acid oxidation turnover. This seems to have been overlooked by the authors.

We critically examined here Chee et al.'s claims that by supplementing daily a small group of older male adults with a carnitine protein formulation, in conjunction with twice‐weekly exercise training sessions over 25 weeks, muscle total carnitine stores would increase and would thereby improve whole‐body insulin sensitivity and increase whole‐body fat (in the form of IMCL) use/oxidation during a subsequent bout of moderate exercise. While the authors correctly rejected the first claim, the present commentator could not find grounds based upon the papers’ results that carnitine supplementation truly increased either (1) muscle‐free carnitine availability or (2) muscle IMCL oxidation during a subsequent moderate‐intensity exercise in older male adults. Thus, the doubts of the commentator are based on the earlier presented calculations and are against the authors’ persistent perspective that total carnitine had the role in the marginal increase in fat oxidation, which was unreasonably accounted for by the rise in muscle IMCL use.

In conclusion, the data reported by Chee et al. are very debatable, and the ‘conclusions’ should be carefully viewed/interpreted considering the shortcomings presently addressed. Before exploring alternative carnitine loading and exercise strategies in older male adults, rather than in the general population, as the authors alluded to, the public would need to be reassured about the validity of the effects of carnitine supplementation on muscle fat oxidation during exercise in older male adults.
